# Synthesis of endohedral fullerenes by molecular surgery

**DOI:** 10.1038/s42004-022-00738-9

**Published:** 2022-10-08

**Authors:** Sally Bloodworth, Richard J. Whitby

**Affiliations:** Chemistry, Faculty of Engineering and Physical Sciences, University of Southampton, Southampton, SO17 1BJ UK

**Keywords:** Synthetic chemistry methodology, Carbon nanotubes and fullerenes

## Abstract

Encapsulation of atoms or small molecules inside fullerenes provides a unique opportunity for study of the confined species in the isolated cavity, and the synthesis of closed C_60_ or C_70_ fullerenes with enclosed atoms or molecules has recently developed using the method of ‘molecular surgery’; in which an open-cage intermediate fullerene is the host for encapsulation of a guest species, before repair of the cage opening. In this work we review the main methods for cage-opening and closure, and the achievements of molecular surgery to date.

## Abstract (web summary)

‘Molecular surgery’ is a useful method through which to create endohedral fullerenes that aren’t accessible by conventional physical methods of trapping small atoms and molecules. Here, the authors review the organic chemistry behind molecular surgery, describing the methods to access open-cage intermediates alongside the cage-closing chemistry.

## Introduction

Endohedral fullerenes (endofullerenes) are stable host-guest complexes in which atoms, ions or molecules are trapped inside the cavity of a fullerene^1^, and are usually sub-grouped as endohedral metallofullerenes^2^ and non-metal endofullerenes^3^.

To date, non-metal endofullerenes in which atomic nitrogen, phosphorus, noble gases, or a small molecule are encapsulated by C_60_ or C_70_ (most commonly) have been prepared, and are denoted A@C_60_ (e.g.,) where ‘A’ represents the trapped endohedral species. They are compounds of enormous interest in several areas; for spectroscopic study of the quantised energy level structure of the trapped species^4–7^, for study of the internuclear (host-guest) interactions resulting from encapsulation and validation of predictive models of these interactions^8,9^, for study of the effect of an encapsulated species upon the properties and reactivity of the cage^10–13^, and for the materials applications that arise in each of these areas. Currently, there are few reviews in these fields, since the availability of non-metal endofullerenes at macroscopic (multi milligram) scale has developed apace only recently.

In early mass spectrometry experiments, collision of accelerated C_60_^+•^ or C_70_^+•^ with helium gas resulted in encapsulation of a single helium atom by the fullerene cage^14–17^, and lead to the development of the first methods for preparation of non-metal endofullerenes by high energy direct insertion. Exposure of C_60_ under high temperature and high pressure of a noble gas, leads to ~0.1% insertion of He, Ne, Ar or Kr, or 0.03% of Xe^18^, and the level of incorporation is improved in the presence of KCN, to 1% for He and ~0.3% for Ar, Kr, or Xe^19–21^. Substantial enrichment of the noble gas content, by removal of empty C_60_ using many cycles of preparative HPLC, is achieved for the heavier endofullerenes, but mass recovery is low (Ar@C_60_, 1.3 mg, 98% filled^21,22^; Kr@C_60_, 1.0 mg, 99% filled^23^; Xe@C_60_ 0.32 mg, 50% filled^20^). Similarly, detection by mass spectrometry of N_2_@C_60/70_, CO@C_60_ and HeNe@C_60_ results from high temperature exposure of the fullerene to a high pressure of the corresponding gas^24^. N_2_@C_60/70_ is also obtained from ion implantation under glow discharge^25^, and the atomic endofullerenes N@C_60/70_ and P@C_60_ have been prepared by ion implantation techniques^25–29^. Pure N@C_60_ has been isolated by exhaustive HPLC enrichment^30^, although the material yield (microgram scale) from these direct insertion methods is too low for many spectroscopic applications^30–32^.

**Fig. 1:**
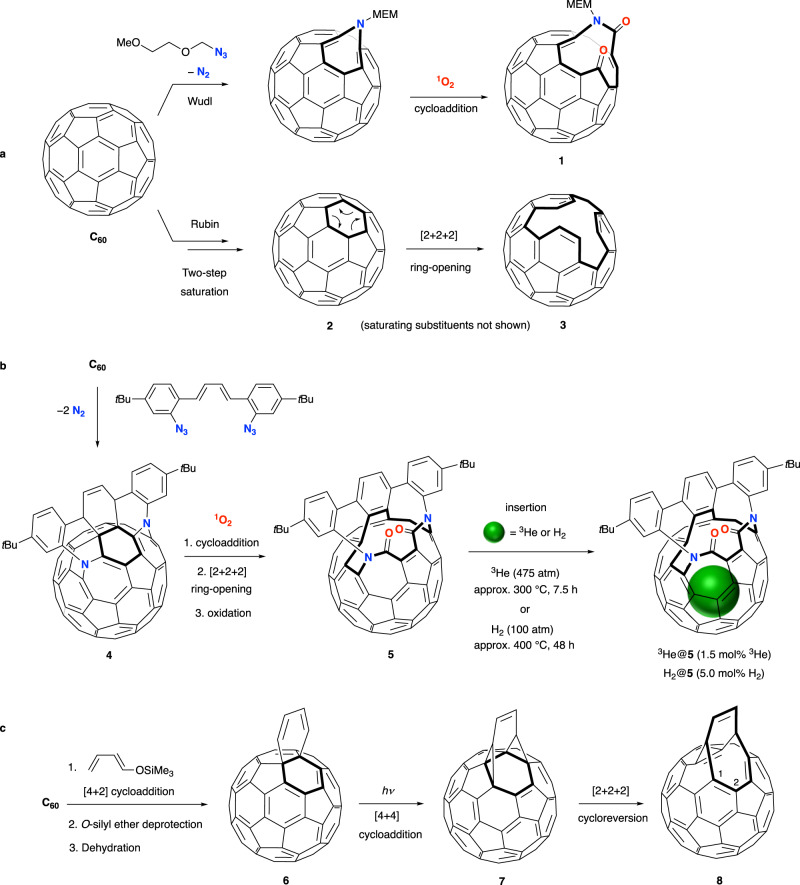
Early studies of C_60_ cage-opening. **a** Wudl’s pioneering one-bond scission and regioselective oxidative cleavage of C_60_^33^, and Rubin’s general strategy to create an opening in C_60_ by two-step saturation of a six-membered ring (saturating substituents on **2** are not shown) followed by [2 + 2 + 2] ring-opening^38^. Open-fullerene **3** has a 15-member orifice, but this elegant route was not realised practically. **b** Controlled partial saturation by a one-pot bis-azide addition to C_60_ in which the fullerene acts as the 2π component in a [4 + 2] Diels-Alder cycloaddition leading to **4**, followed by cycloaddition of ^1^O_2_ and [2 + 2 + 2] ring-opening. Bis-lactam **5** has an orifice that allows entry of He or H_2_ into the cage^41^. **c** Cycloadduct **6** undergoes photochemically induced [4 + 4] rearrangement, before thermal [2 + 2 + 2] cycloreversion of **7**, an unstable intermediate^42^. The isolable product **8** is of a general structure common to all subsequent reports of C_60_ cage-opening that have been applied for synthesis of non-metal endofullerenes A@C_60_.

In the mid 1990s, Fred Wudl and Yves Rubin proposed the synthesis of endofullerenes from a multi-step procedure in which an opening in the fullerene is created, of suitable size to allow entry of a guest species into the cavity before a series of reactions to repair the cage-opening—restoring the original fullerene with the endohedral guest species trapped inside^33,34^. For realisation of this approach, controlled methods to create an opening in the fullerene cage would be required and Wudl accomplished the earliest cage-opening of C_60_ by 1,3-dipolar cycloaddition of an alkyl azide—involving scission of a single bond of C_60_ (Fig. 1a). Furthermore, controlled expansion of the orifice size was demonstrated by a contiguous oxidative cleavage, *via* [2 + 2] cycloaddition with singlet oxygen^33,35^. A strategy of two-step saturation of a six-membered ring of the fullerene cage, followed by [2 + 2 + 2] ring-opening, was explored by Rubin (Fig. 1a)^36–38^, after both authors showed that C_60_ participates as the 2π component in [4 + 2] cycloadditions (achieving partial saturation, a good first step)^39,40^. Interruption of the intended sequence by reaction of the first cycloadduct, **4**, with ^1^O_2_ lead to an open-cage derivative (**5**) of C_60_ with the largest orifice then known, able to accommodate H_2_ (5 mol%) or an atom of ^3^He (1.5 mol%) when heated under a high pressure of either gas (475 atm ^3^He, or 100 atm H_2_) (Fig. 1b)^41^.

Preparation of these first ‘open’ endohedral fullerenes, H_2_@**5** and ^3^He@**5**, was just one of two early milestones achieved by the Rubin group. In 1996, the product, **6**, of Diels-Alder [4 + 2] cycloaddition between C_60_ and 1-((trimethylsilyl)oxy)-1,3-butadiene, acidic cleavage of the silyl ether and dehydration, was found to undergo photochemical [4 + 4] rearrangement followed by [2 + 2 + 2] cycloreversion of the unstable intermediate **7** (Fig. 1c)^42^. The isolable ethene-bridged product **8** has an eight-membered opening too small for the entry of guest species into the cavity. Importantly however, this sequence of a Diels-Alder cycloaddition of C_60_ coupled with elimination to form an intermediate of core structure **6**, followed by the [4 + 4] and [2 + 2 + 2] pericyclic steps, would become the initial cage-opening process of all subsequent syntheses of non-metal endofullerenes—every example of which involves an intermediate of general structure **8** as the first stable C_60_ (or C_70_) derivative in the reaction sequence.

Herein we give a succinct review of the synthesis of non-metal closed endohedral fullerenes via open-cage intermediates, routes that have become known as ‘molecular surgery’. In our discussion of open-fullerenes, we include only those for which encapsulation of an atom or molecule has been demonstrated, and we further limit these to the examples whose synthesis contributed methods to the development of completed routes to A@C_60/70_. An excellent, comprehensive review of open-cage fullerenes is already available^43^. More recent studies of the encapsulation of small molecules by open-fullerenes derived from fullerene-mixed peroxides, towards applications of the *open* host-guest complex by selective trapping/release of the guest, were conducted by the group of Liangbing Gan and lately reviewed^44^.

Synthesis of closed endofullerenes will be categorised according to two main synthetic routes for encapsulation of (i) ‘small’ guest species He, Ne, H_2_, HF or H_2_O in the fullerene cage, and (ii) all larger noble gas atoms and small molecules. Current challenges are discussed in a final ‘outlook’ section.

### Open-cage fullerenes

Concurrent methods to obtain an open-fullerene with core structure **8** in one-pot from C_60_, followed by regioselective oxidative cleavage to widen the cage-opening, were developed by the groups of Shizuaki Murata^45,46^ and Koichi Komatsu^47–49^.

**Fig. 2:**
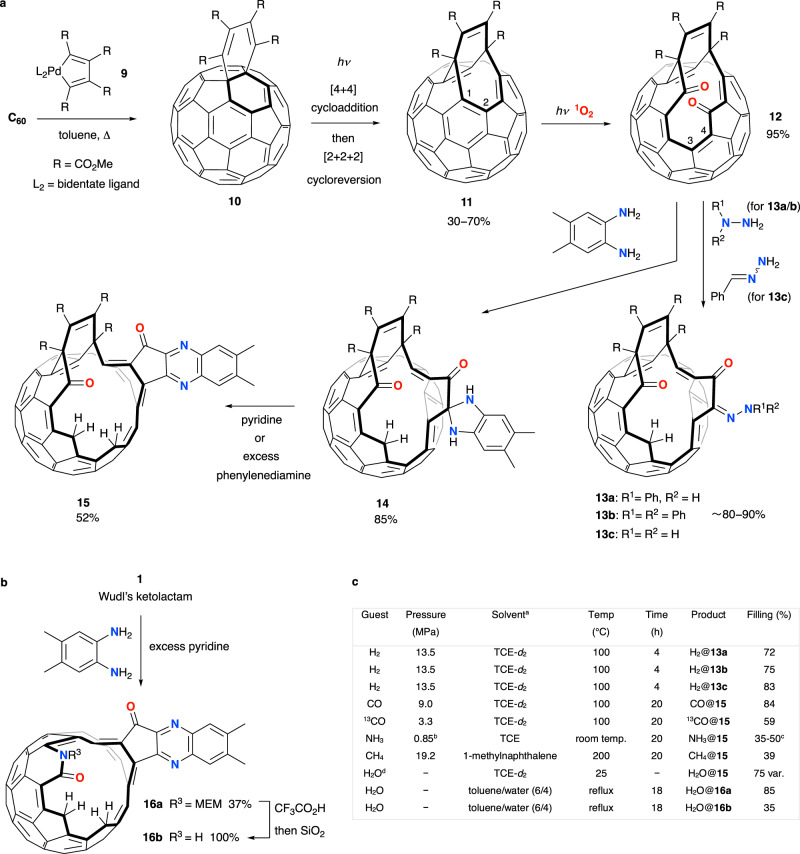
Iwamatsu open-cage derivatives of C_60_ and their encapsulation of molecular species. **a** An open-fullerene (**11**) with the core ethene-bridged structure of **8** is obtained in one-pot from C_60_, and undergoes regioselective oxidative cleavage. Widening of the opening is achieved by further reaction with a hydrazine or 1,2-diamine. **b** Wudl’s ketolactam **1** is also a substrate for orifice-widening with an *o*-phenylenediamine. **c** Conditions for molecular guest encapsulation by open-fullerenes **13**, **15** and **16**; ^a^TCE = 1,1,2,2-tetrachloroethane, ^b^Approx. 0.85 MPa (i.e., vapour pressure of NH_3_ at room temp.), ^c^Partial loss of NH_3_ occurs slowly, during 6 months storage at −10 °C, ^d^H_2_O encapsulation by **15** occurs under ambient pressure and the % filling shows a temperature-dependent entry/escape equilibrium (var. = variable).

Murata reported the cycloadduct (**10**) of Diels-Alder reaction between C_60_ and palladacyclopentadiene complexes, **9**, to undergo photoinduced [4 + 4] rearrangement and [2 + 2 + 2] cycloreversion, identically to the Rubin sequence, to give open-fullerene **11** in ~70% yield (based upon **9** = dimethoxyglyoxime complex, Fig. 2a)^46^. The HOMO of ethene-bridged open-fullerenes with core structure **8** is localised at the (two) double bond(s) C(1)-C(2)^48^, and oxidative cleavage of **11** occurs regiospecifically at this position upon irradiation of a toluene or CHCl_3_ solution of **11** in air, since formation of ^1^O_2_ is sensitised by the fullerene itself^45^. The resulting diketone, **12**, has too small an opening for the entry of a guest molecule but was found to undergo an unusual reaction with either substituted hydrazines or hydrazones^50^, or *o*-phenylenediamine^51^ reagents—each involving clean and highly selective scission of the C(3)-C(4) bond by hydroamination. Open-fullerenes, **13** and **14** were obtained respectively, and **14** was found to participate in further selective widening of the cage in the presence of additional amine base. Similarly, widening of the orifice of Wudl’s ketolactam open-fullerene **1** was achieved with *o*-phenylenediamine and excess pyridine, to furnish **16** (Fig. 2b)^52^. S-i. Iwamatsu and S. Murata have reviewed the elegant characterisation work which they carried out to elucidate the structures of **13**–**16**^53^, and demonstrated the encapsulation of molecular guests (Fig. 2c). Encapsulation of molecular hydrogen by open-fullerenes **13a**-**c** occurs under 13.5 MPa H_2_ at 100 °C, with up to 83% ‘filling’ of the cage^50^. The openings of **15** or **16** are too large to prevent the rapid escape of H_2_, but allow larger guest species to be accommodated and retained. Water enters both **15** and **16a**/**b** under ambient conditions, and temperature-dependent loss of water from H_2_O@**16a**/**b** is much slower than from H_2_O@**15** which has a bigger cage opening^51,52^. Accordingly, encapsulation of CO, NH_3_ and CH_4_ by **15** was accomplished under conditions of moderate pressure to furnish stable endohedral fullerene products, of which NH_3_@**15** is reported to undergo a (slow) partial loss of the guest molecule^54–56^.

**Fig. 3:**
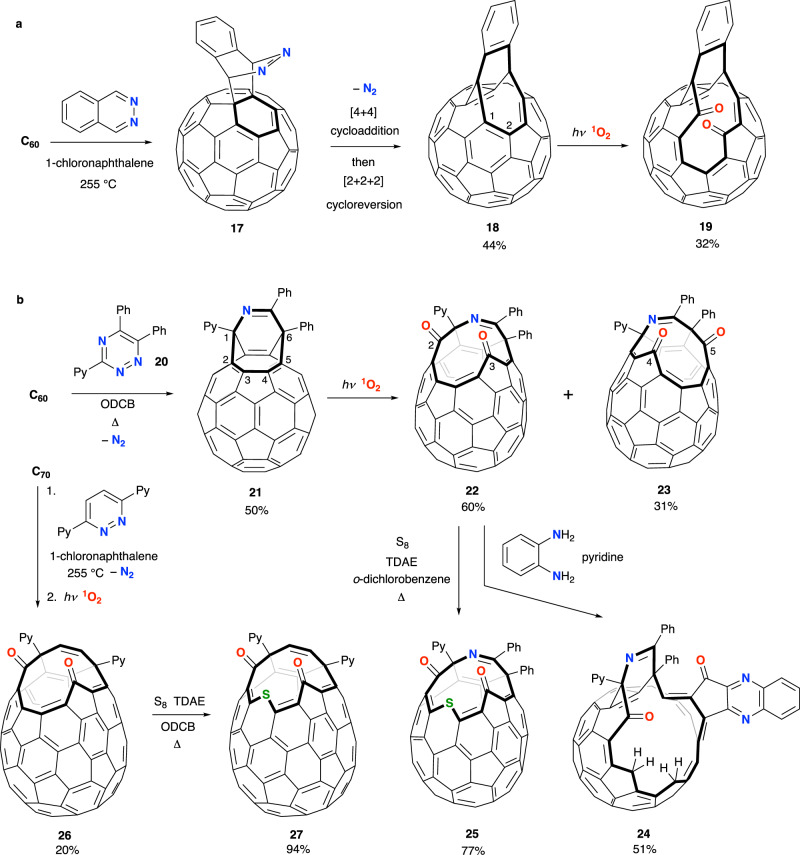
Open-fullerenes prepared from reaction of C_60_ with pyridazine derivatives. **a** An open-fullerene (**18**) with the core ethene-bridged structure of **8** is obtained in one-pot from C_60_, and undergoes regioselective oxidative cleavage. **b** Cycloaddition of C_60/70_ with a substituted triazine or pyridazine. Abbreviations: 2-pyridyl (Py), *o*-dichlorobenzene (ODCB), tetrakis(dimethylamino)ethylene (TDAE).

Akin to the pathway described by Murata and Iwamatsu, a further example of one-pot preparation of an open C_60_ derivative with core structure **8** was reported by Komatsu (Fig. 3). Upon heating C_60_ with phthalazine, in 1-chloronaphthalene solution near reflux, open-fullerene **18** was obtained from [4 + 2] cycloaddition, loss of N_2_ from an unstable intermediate **17**, and the [4 + 4] addition / [2 + 2 + 2] reversion sequence already described^47^. Oxidative cleavage of C(1)-C(2) is regiospecific, although diketone **19** was obtained in modest yield (Fig. 3a)^48^ cf. the comparable diketone **12**. Further examples of [4 + 2] cycloaddition between C_60_ or C_70_ (as the 2π component) and ‘diene’ partners embedded in a pyridazine core structure, like the reaction with phthalazine, would lead to the general adoption of this method for the initial cage-opening in synthesis of endofullerenes. These have been reviewed recently^57^. Of the first examples (Fig. 3b), a substituted 1,2,3-triazine, **20**, was partnered with C_60_ to confer solubility of the open-fullerene product(s) in common organic solvents^58^. The cycloadduct **21** is an imine-bridged asymmetric analogue of the ethene-bridged compounds **8**, **11** or **18** and, from DFT calculations, the HOMO of **21** is localised at the C(2)-C(3) and C(4)-C(5) double bonds (similarly to the ethene-bridged examples). Accordingly, oxidative cleavage of either the C(2)-C(3) or C(4)-C(5) double bond of **21** using ^1^O_2_ leads to a separable mixture of **22** and **23**, respectively. The major product, **22**, was obtained in 61% yield and its orifice can be widened by: (i) Iwamatsu’s regioselective addition of an aromatic hydrazine or hydrazone^59^, or *o*-phenylenediamine^60^ to give **24**; or, (ii) sulfur atom insertion using S_8_ in the presence of a single-electron reductant, tetrakis(dimethylamino)ethylene (TDAE), from which **25** is isolated in good yield^58^.

The cage-opening of **24** is of the same size as its all-carbon analogue **15**, and **24** was similarly shown to encapsulate water under conditions of ambient pressure. The equilibrium between empty **24** and H_2_O@**24** is dependent upon temperature and solvent polarity. Encapsulation of formaldehyde or HCN by **24** also occurs under conditions of ambient pressure; H_2_CO@**24** is observed as the minor component (9%) in an inseparable mixture with H_2_O@**24** (35%) and empty **24** (56%) by passing gaseous formaldehyde through a solution of **24** in chlorobenzene at 100 °C. Treatment of a mixture of **24** and H_2_O@**24** in chlorobenzene with excess HCN at 90 °C results in displacement of water and recovery of HCN@**24** with near-quantitative HCN incorporation. Slow thermal dissociation of HCN@**24** is reported^60^.

Steps to repair the cage-opening of the endohedral open fullerenes derived from the hydroamination reactions (A@**13**, A@**15**, A@**16**, A@**24**) have not been developed, as it is a hugely challenging task to find conditions for reversal of the complex rearrangement steps that follow the initial amine condensation. Instead, reversal of the route by which open-fullerene **25** is prepared i.e., by sulfur extrusion and a McMurry-type reductive coupling of the diketone as first steps, is a practicable approach and would be explored by Komatsu and co-workers (see ‘Synthesis of closed endohedral fullerenes’ below). Of course, it is necessary that the orifice of **25** is big enough for the entry of a guest species, and although the 13-membered ring is smaller than that of any example discussed above (**5**, **13**, **15**, **16** or **24**) calculation of the activation barrier to entry of small guests He and H_2_ into **25** (18.9 and 30.1 kcal mol^−1^ respectively)^61^ with that for entry to **5** (24.5 and 41.4 kcal mol^−1^ respectively)^41^ suggests that encapsulation in **25** could be achieved. Indeed, upon heating a powdered sample of **25** at 200 °C under 800 atm of H_2_ a quantitative recovery of H_2_@**25** was made. The experimental activation energy for escape of hydrogen from H_2_@**25** is *E*_a_ = 34.2 ± 0.58 kcal mol^−1^, and so the complex is stable to dissociation at room temperature^61,62^. The barrier to escape of helium from ^3^He@**25** (*E*_a_ = 22.8 kcal mol^−1^)^63^ is much lower than that for dissociation of H_2_@**25**, so after heating **25** at 90 °C under 650 atm of helium, >35 mol% encapsulation is inferred by cooling He@**25** to −20 °C and reduction with NaBH_4_ to form a hemiaminal ether across the cage-opening that blocks the escape^64^.

A closely alike sequence to that used for preparation of **25** was adopted to obtain **27**, an open-cage derivative of C_70_ (Fig. 3b). Thermal cycloaddition between C_70_ and 3,6-di(2-pyridyl)pyridazine, then photooxidation under xenon lamp irradiation in air, lead to **26** before sulfur insertion afforded **27**. The calculated energy barrier for encapsulation of H_2_ in the cavity of **27** is 31.2 and 31.0 kcal mol^−1^ for entry of a first then second molecule, respectively (cf. 30.1 kcal mol^−1^ for H_2_ entry into **25**), and suggests that the 13-membered cage-opening is of comparable size to that of **25**—as might be expected from their structural resemblance. Accordingly, heating a powdered sample of **27** at 200 °C under 830 atm of H_2_ gave a mixed sample of H_2_@**27** (97%) and (H_2_)_2_@C_70_ (3%)^65^.

### Synthesis of closed endohedral fullerenes A@C_60_ and A@C_70_

#### Synthesis of closed fullerenes containing small guest species, He, Ne, H_2_, HF or H_2_O

**Fig. 4:**
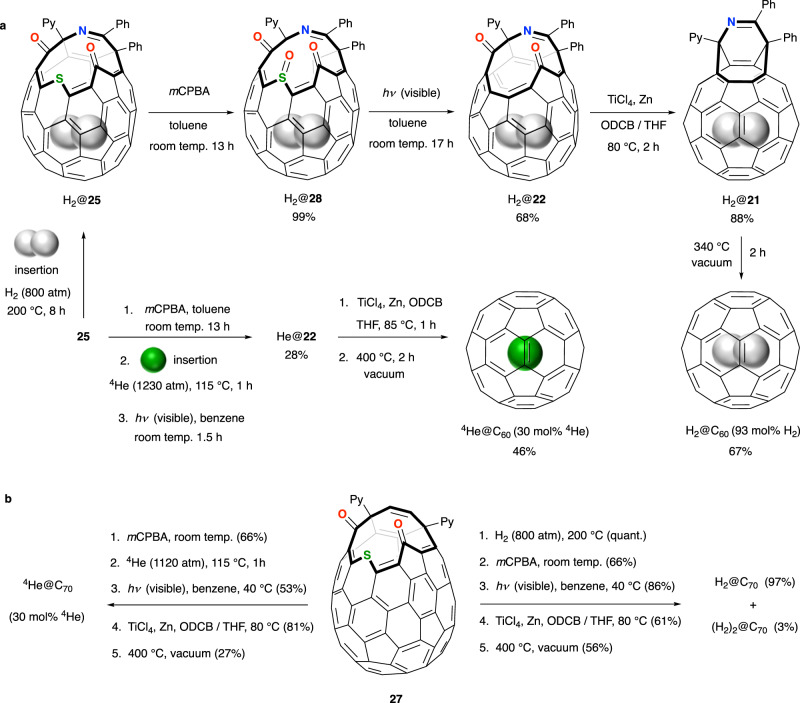
Komatsu’s synthesis of H_2_@C_60/70_ and ^4^He@C_60/70_. **a** Synthesis of H_2_@C_60_ and ^4^He@C_60_ *via* sulfide oxidation and photochemical desulfinylation, each at ambient temperature. The order of steps is altered to avoid an intermediate ^4^He@**25** from which escape of helium is very facile. **b** Synthesis of H_2_@C_70_ and ^4^He@C_70_ using identical methods. Tetrahydrofuran (THF), 2-pyridyl (Py), *o*-dichlorobenzene (ODCB).

When Komatsu’s open-cage endofullerene H_2_@**25** was subjected to matrix-assisted laser desorption/ionization time-of-flight (MALDI-TOF) mass spectrometry, molecular ion peaks for H_2_@**25** (*m*/*z* 1068), empty **25** (*m*/*z* 1066), H_2_@C_60_ (*m*/*z* 722) and C_60_ (*m*/*z* 720) were observed; indicating that repair of the cage-opening of H_2_@**25** to obtain H_2_@C_60_ is feasible although substantial loss of H_2_ (~70%) occurs with gas-phase laser irradiation^61^. The half-life for thermal dissociation of H_2_@**25** is *t*_1/2_ = 54.4 h at 160 °C, but the complex is stable at room temperature so reaction conditions to reduce the size of the cage-opening must avoid high-temperatures. This was accomplished by the Komatsu group in their landmark synthesis of H_2_@C_60_, using methods they also applied for the synthesis of ^4^He@C_60_ and shown in Fig 4a^66–68^. Oxidation of H_2_@**25** at room temperature gives *exo*-sulfoxide H_2_@**28**, more stable than the *endo*-sulfoxide by 8.6 kcal mol^−1^ presumably as a result of steric congestion between the sulfinyl and carbonyl groups in the *endo*-form^68^. Then, constriction of the size of the opening is also achieved under very mild conditions, by a photochemical desulfinylation under visible light irradiation. The contracted product of SO extrusion, H_2_@**22**, is thermally stable—no loss of H_2_ occurs after heating a solution of H_2_@**22** in 1,2-dichlorobenzene-*d*_4_ at 190 °C for 3 days, *cf. t*_1/2_ = 4.2 h for thermal dissociation of H_2_@**25** at 190 °C. Correspondingly, McMurry reductive coupling of H_2_@**22** was performed without loss of endohedral hydrogen at 80 °C, returning to the imine-bridged intermediate H_2_@**21**, before final closure of the cage was accomplished by heating at 340 °C under vacuum. H_2_@C_60_ is thereby obtained with 93 mol% encapsulation of H_2_ in an overall yield of 9% from C_60_, and pure H_2_@C_60_ was recovered after preparative recycling HPLC in a substantial material quantity of ~100 mg^66,68^.

As helium escapes rapidly from He@**25** at room temperature (in previous work, this species was reduced to a hemiaminal ether derivative in which the cage-opening is blocked, i.e., helium is trapped, see earlier) it proved necessary to re-order the reaction sequence described for synthesis of H_2_@C_60_, in order to prepare He@C_60_ (Fig. 4a). Oxidation of empty open-fullerene **25** was carried out first, and the sulfoxide derivative **28** was then the substrate for ‘filling’ under 1230 atm of helium gas at 115 °C before cooling to ambient temperature whilst pressurised, and rapid photo-desulfinylation for just 1.5 h, at room temperature. Encapsulation of endohedral species (e.g., ^4^He in this example) under high-pressure conditions well in excess of 1200 atm is cited in many further examples, vide infra, and although we refer the reader to the original literature for details of the bespoke apparatus used therein, typically relies upon hydraulic or manual gas compression following initial pressurisation. ^4^He@**22** was obtained with 30 mol% incorporation of helium and no loss of the endohedral atom occurred during the final two steps, McMurry reductive coupling and thermal closure. Enrichment to a sample of ^4^He@C_60_ with 95% helium encapsulation was achieved^67^.

These cage-closure methods were also applied for the preparation of a separable mixture of H_2_@C_70_ + (H_2_)_2_@C_70_ that were each isolated as the pure endofullerene^69^, and ^4^He@C_70_ with 30 mol% helium incorporation^67^ (Fig. 4b). Helium incorporation was enriched to 60 mol% by recycling HPLC. The endohedral helium dimer was not detected, although it is known that the cavity of C_70_ can accommodate two helium atoms^70^.

**Fig. 5:**
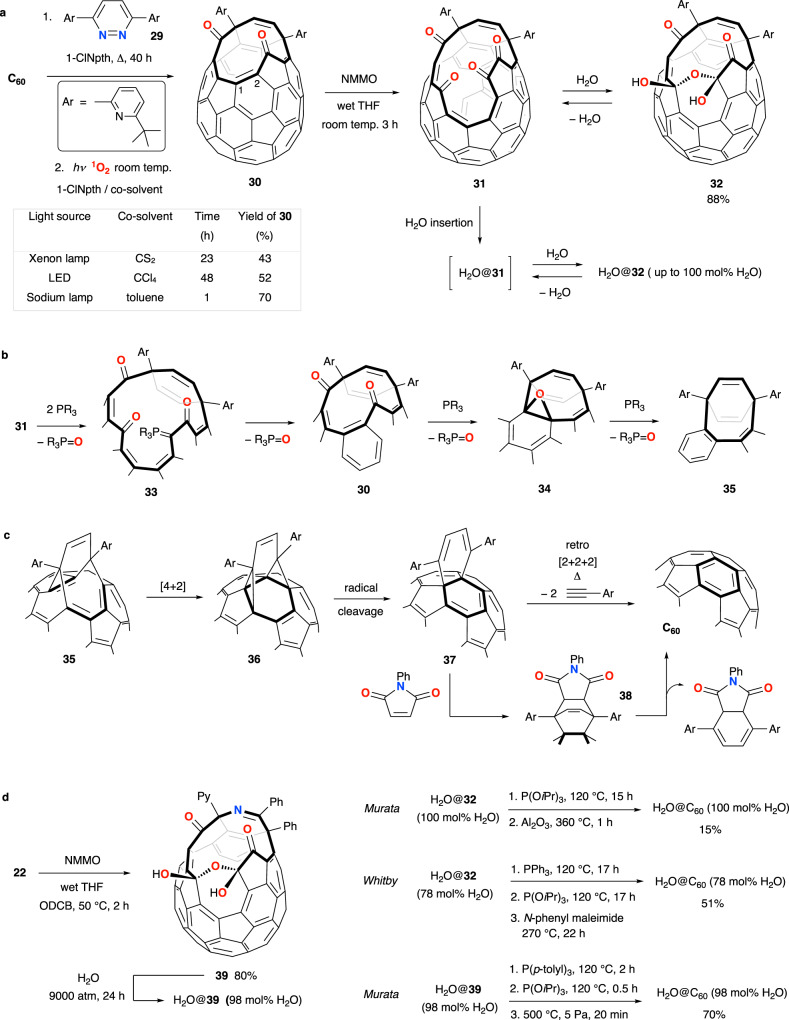
Synthesis of H_2_O@C_60_. **a** Open-cage fullerene **31** has a 16-membered orifice and is the substrate for encapsulation of H_2_O. Encapsulation occurs by in situ dehydration of **32**, see the main text for detail. **b** Double reductive coupling of carbonyl groups on the 16-membered tetraketone cage-opening. Only the orifice atoms are shown. **c** Final cage-closure. **d** Synthesis of H_2_O@C_60_. 1-chloronaphthalene (1-ClNpth), *N*-methylmorpholine *N*-oxide (NMMO), 2-pyridyl (Py), *o*-dichlorobenzene (ODCB).

The molecular endofullerene H_2_O@C_60_ is an important target for synthesis by molecular surgery, as a substrate for study of the rich, quantum energy level structure of the isolated water molecule. In order to achieve the synthesis of H_2_O@C_60_, an open-cage derivative of C_60_ with a larger opening than the examples discussed so far was required (Fig. 5). After cage-opening of C_60_ by reaction with 3,6-bis(6-(*tert*-butyl)pyridin-2-yl)pyridazine (**29**) according to the now well-established sequence of [4 + 2] cycloaddition, [4 + 4] rearrangement and retro-[2 + 2 + 2] cycloreversion, photo-oxidative cleavage leads to diketone **30**^71^. The first report of this reaction with ^1^O_2_ required irradiation of a solution of the open-fullerene in mixed 1-chloronaphthalene and CS_2_ solvents for 23 h, as oxygen is passed through the reaction vessel. However, CS_2_ has flashpoint ca. 30 °C and an auto-ignition temperature of 100 °C, prompting the research groups of both Yasujiro Murata and Richard Whitby to seek safer alternatives; CS_2_ may be replaced with CCl_4_ under LED irradiation to obtain **30** in 52% yield^72^, and switching the co-solvent to toluene leads to the isolation of **30** in an improved yield of 70% after just 1 h under irradiation with a high-pressure sodium lamp (Fig. 5a)^73^. The isolated yield of **30** is measured over two steps in which the C_60_ starting material is present in excess—i.e., acting as photosensitizer in the singlet oxygenation. Murata and co-workers found that a second regiospecific oxidative cleavage of the C(1)-C(2) bond of **30** takes place using *N*-methylmorpholine *N*-oxide, and the resulting tetraketone, **31**, is isolated as its bis(hemiketal) hydrate **32** when the oxidation is carried out in wet THF. The 16-membered cage opening of **31** is large enough for entry of water, so upon heating a solution of **32** in wet toluene at 120 °C, for 36 h under 9000 atm, quantitative recovery of H_2_O@**32** is made via the dynamic equilibrium between **31** and **32**, that enables encapsulation of H_2_O by **31** whilst the water molecule is unable to escape from the hydrate **32**, since the 13-membered opening is too small^71^. Without pressurisation, a solution of **32** in wet toluene equilibrates to 23 mol% endohedral water content after 36 h at 120 °C; and Whitby et al. found that heating a solution of **32** in 1-chloronaphthalene with water to 100 °C in a sealed tube gives H_2_O@**32** with 78 mol% endohedral water, after 48 h (i.e., under some pressurisation but using accessible conditions that require no special apparatus)^74^.

Repair of the cage-opening of **32** involves dehydration to return to the tetraketone **31**, then sequential reductive couplings of the ‘paired’ carbonyl groups that were formed in the sequential oxidative cleavage steps during cage-opening (Fig. 5b). Reductive coupling is achieved upon reaction with trivalent phosphorus reagents, by a mechanism that involves initial formation of an intermediate β-oxo-phosphorus ylid **33**, then intramolecular Wittig reaction that returns the cage-opening to the diketone **30**^73,75^. Formation of the β-oxo-phosphorus ylid **33** could take place via attack of phosphorus at the carbonyl carbon followed by [1, 2]-phospha-Brook rearrangement, by electron transfer to the fullerene then attack of phosphorous directly at oxygen, or by Kukhtin-Ramirez addition^76–78^; elimination of R_3_P = O occurs with another phosphine/phosphite addition in each case. Diketone **30** is then subject to the same reduction sequence, although calculations support the formation of an epoxide intermediate **34** from the first +PR_3_/−R_3_P = O step, rather than a mechanism involving phosphorus ylid formation and intramolecular Wittig reaction^79^. With an excess of the phosphorus reagent, the stable ethene-bridged derivative **35** is obtained. The final step of the closure sequence leads to C_60_, and involves sequential [4 + 2] intramolecular cycloaddition, radical cleavage of the strained intermediate **36** (formally a retro [4 + 4] cycloaddition) and [2 + 2 + 2] cycloreversion (Fig. 5c)^68^. In their syntheses of ^4^He@C_60_, H_2_@C_60_, H_2_@C_70_ and (H_2_)_2_@C_70_ described earlier (Fig. 4), Komatsu and Murata employed vacuum pyrolysis for this step.

So, from their sample of pure H_2_O@**32** obtained by high-pressure filling, the Murata group achieved the first synthesis of H_2_O@C_60_—effecting dehydration to H_2_O@**31** and the sequential reductive couplings with excess P(O*i*Pr)_3_ in refluxing toluene, before vacuum pyrolysis of alumina-supported solid H_2_O@**35** to complete the closure. H_2_O@C_60_ was obtained in 15% over these steps (Fig. 5d)^71^.

The yield of the first reductive coupling (of H_2_O@**31**) using alkyl phosphite reagents is compromised by unwanted formation of an α-hydrophosphate side-product, but clean reduction occurs with trialkyl phosphines^74^. From their sample of H_2_O@**32**, with 78 mol% endohedral H_2_O, the Whitby group carried out dehydration of the bis(hemiketal) to obtain H_2_O@**31** with in situ clean reduction to H_2_O@**30** using excess PPh_3_ in refluxing toluene. The second reductive coupling was then conducted with P(O*i*Pr)_3_, and the final pyrolysis was adjusted to follow a lower energy pathway in the presence of *N*-phenylmaleimide^39^, which reacts with intermediate **37** in a [4 + 2] Diels-Alder reaction. The cycloadduct **38** reverts to C_60_ via a retro [4 + 2] cycloaddition (Fig 5c) and H_2_O@C_60_ (78 mol% H_2_O) was obtained in an improved yield of 51% from H_2_O@**32** (Fig. 5d)^74^.

Finally, to optimise the synthesis of H_2_O@C_60_, Murata recently reported theoretical modelling of water encapsulation and cage closure steps for structural analogues of tetraketone **31**, choosing substituent patterns around the orifice that could be readily accessed according to the choice of azine used as the ‘diene’ 4π partner to C_60_ (the 2π component) in the first [4 + 2] pericyclic cage-opening step of the molecular surgery route. The optimal open-cage derivative was predicted to be the one formed from oxidative cleavage of Komatsu’s diketone **22** (Fig. 3), which was therefore prepared from **22** using *N*-methylmorpholine *N*-oxide in wet THF, and isolated as its bis(hemiketal) hydrate **39**. Near-quantitative water encapsulation was achieved under high-pressure to give H_2_O@**39** (i.e., similarly to high-pressure quantitative water ‘filling’ of **32** via the equilibrium with its dehydrated form), and in situ dehydration of the bis(hemiketal) then sequential reductions with P(*p*-tolyl)_3_ and (P(O*i*Pr)_3_ were effected in a single pot, before vacuum pyrolysis gave H_2_O@C_60_ (98 mol% H_2_O) with 87% isolated yield in the pyrolysis step and in 70% from H_2_O@**39** (Fig. 5d)^79^.

The same methods have also been applied for synthesis of H_2_O@C_70_. Initial [4 + 2] Diels-Alder cycloaddition of 3, 6-bis(6-(*tert*-butyl)pyridin-2-yl)pyridazine (**29**) occurs at the α-bond or β-bond of ellipsoidal C_70_ to yield isomeric products in 42% and 6% (from α- and β-bond scission, respectively) after the 4 + 4] rearrangement and retro-[2 + 2 + 2] cycloreversion sequence. Widening of the cage-opening of each isomer can be achieved using the methods already described; photo-oxidative cleavage with ^1^O_2_ followed by a second regiospecific oxidative cleavage with wet 4-dimethylaminopyridine *N*-oxide yields isomeric tetraketone open-fullerene derivatives of C_70_ each with a cage-opening of identical structure to the C_60_ analogue **31**. However, only the C_70_ open-fullerene tetraketone derived from initial scission of the β-bond is easily able to accommodate entry of water, presumably since there is more strain release associated with β-bond scission (or a C_60_ bond scission) which corresponds to a bigger resultant orifice^80^. Correspondingly, the β-bond scission isomer is the minor product of C_70_ cage-opening, but is the required intermediate to achieve the encapsulation and closure steps. These have been carried out as already discussed for synthesis of H_2_O@C_60_ (Fig. 5); i.e., water uptake in wet toluene at 120 °C, for 40 h under 9000 atm, double reductive coupling with excess P(O*i*Pr)_3_ in refluxing toluene, and thermal closure in the presence of *N*-phenylmaleimide. Pure H_2_O@C_70_ is obtained by preparative single-stage HPLC, from a separable mixture containing *ca*. 18% empty C_70_ and trace (H_2_O)_2_@C_70_^81^. Interestingly, encapsulation of H_2_O into the C_70_ open-fullerene tetraketone derived from initial α-bond scission does occur in the presence of HF; treatment with wet HF-pyridine (70% *w*/*w*, 0.5 molar equiv.) at 9000 atm. and 120 °C for 18 h, results in encapsulation of HF (32 mol%), H_2_O･HF (11 mol%) and H_2_O (27 mol%). After cage closure, pure (H_2_O･HF)@C_70_ can be isolated from an inseparable mixture of H_2_O@C_70_ and HF@C_70_^82^.

**Fig. 6:**
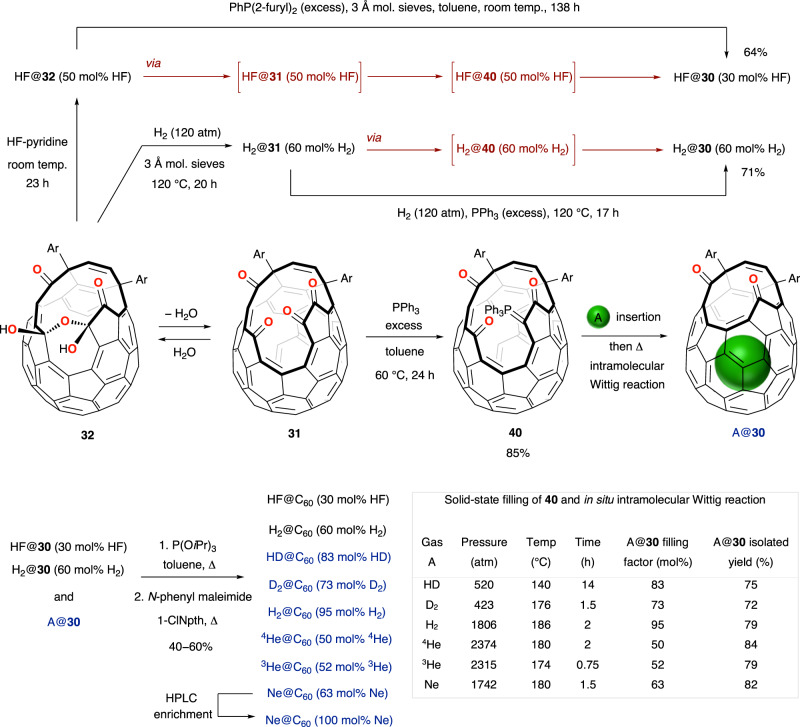
Whitby’s synthesis of closed C_60_ endofullerenes containing a small endohedral species. Open-cage fullerenes **31** and **40** each have a 16-membered orifice able to accommodate the entry of guest species. Solution-phase encapsulation of H_2_ or HF by **31** occurs *via* dehydration of bis(hemiketal) **32** for synthesis of H_2_@C_60_ and HF@C_60_. Solid-state filling of **40** was performed under the tabulated conditions for optimised synthesis of H_2_@C_60_ and He@C_60_ isotopologues, and Ne@C_60_. High-pressure solid-state filling was carried out in a 100 × 5.2 mm 316 L stainless steel reactor as part of a bespoke apparatus for gas compression using a manual pump.^73^ 1-Chloronaphthalene (1-ClNpth), the ‘Ar’ 5-*tert*-butylpyridyl substituent structure is shown in Fig. 5a.

Calculation of the binding energy and activation energies of entry/exit for encapsulation of guest species into open-cage fullerenes is a vital tool to inform the conditions of ‘filling’ and subsequent steps, towards synthesis of A@C_60_. This approach has been applied for syntheses of H_2_@C_60_, HF@C_60_ and the smaller noble gas endofullerenes He@C_60_ and Ne@C_60_ (Fig. 6).

The calculated activation energies for both the entry of HF into **31**, and its loss from the cage, indicate that encapsulation and release of HF are much more favourable than the corresponding trapping/release of H_2_O by the same open-fullerene^75,83^. So, an optimal 50 mol% filling with HF occurs under ambient conditions, by equilibration of a solution of either **31** or **32** in CH_2_Cl_2_ with excess HF-pyridine at room temperature^83^. Conversion of HF@**32** to HF@**31** occurs simply by stirring with molecular sieves at room temperature, and the β-oxo-phosphorous ylid intermediate of the first reductive coupling closure step, HF@**40**, is isolated from slow reaction between HF@**31** and PPh_3_—also at ambient temperature. However, the intramolecular Wittig reaction of HF@**40** which completes the reduction step requires heating to >100 °C and causes complete thermal dissociation. Loss of HF is minimised using di-(2-furyl)phenylphosphine which effects the reduction of HF@**31** at a lower temperature, hence HF@**30** is obtained in good yield with 30 mol% remaining HF incorporation from HF@**32** (50 mol% HF) (Fig. 6). No loss of HF takes place from the small (12-membered) cage-opening of HF@**30**, so the second reduction is safely carried out with P(O*i*Pr)_3_ in refluxing toluene, and thermal closure in the presence of *N*-phenylmaleimide returns HF@C_60_ (30 mol% HF)^75^.

The calculated activation energy for entry of H_2_ into the cavity of **31** is *ca*. 12 kJ mol^−1^ higher than that for entry of H_2_O^83^, despite the smaller size of H_2_ and presumably due to the attractive dipolar interactions of H_2_O in the cage entrance. Yet, as substantial H_2_O incorporation into **31** is achieved under very mild conditions (78 mol% using wet 1-chloronaphthalene at 100 °C in a sealed tube—see earlier^74^), Whitby showed that 60 mol% encapsulation of H_2_ in **31** takes places under conditions of only moderate pressure—under 120 atm H_2_ at 120 °C bis(hemiketal) **32** undergoes in situ dehydration, accelerated with molecular sieves, to form H_2_@**31**. Heating H_2_@**31** with PPh_3_ then induces a contraction of the cage-opening by the first reductive coupling, but has to be conducted under the same H_2_ pressurisation to avoid loss of the endohedral molecule (Fig. 6). A second reduction with P(O*i*Pr)_3_, then thermal closure in the presence of *N*-phenylmaleimide, completed the synthesis of H_2_@C_60_ (60 mol% H_2_) in 51% yield from **31**^74^.

This route complements the synthesis of H_2_@C_60_ by Komatsu (Fig. 4), although H_2_@C_60_ was obtained with 93 mol% filling via Komatsu’s more forcing conditions for H_2_ encapsulation by **25** (800 atm H_2_ at 200 °C). After showing that the β-oxo-phosphorus ylid **40** is an isolable intermediate Whitby surmised that, if entry of H_2_ (or another species) into **40** could occur at a temperature lower than that required for the following intramolecular Wittig reaction, it would be possible to ‘fill’ the phosphorus ylid **40** then induce the Wittig closure that traps the endohedral species simply by raising the temperature^73^. Calculation of the activation enthalpies for entry of small guests, H_2_, He and Ne, through the 16-membered openings of **31** and **40** indicates that—in each case—the barrier to entry into **40** is only ca. 10 kJ mol^−1^ higher than that for entry into **31**, and it was found that ‘closure’ of H_2_@**40** (to H_2_@**30**) occurs after equilibration of H_2_ between the fullerene cavity and outside. As the Wittig reaction is unimolecular (cf. overall reductive coupling of **31**) it also became possible to conduct the combined encapsulation and Wittig reaction steps without solvent, with significant advantages—a small (ca. 1–5 mL volume) pressure reactor can be used, so that high-pressure conditions can be safely achieved whilst the volume of gas remains low, allowing rare and/or expensive gases to be used. So, solid-state filling of **40** with the isotopologues of molecular hydrogen and helium, H_2_, HD, D_2_, ^3^He and ^4^He, as well as with Ne, was achieved with in situ thermal contraction of the cage-opening according to the conditions of Fig. 6 (table). Cage-closure of the resulting diketone endofullerenes A@**30** (A = H_2_, HD, D_2_, ^3^He, ^4^He or Ne) was carried out using the usual conditions of a reductive coupling with P(O*i*Pr)_3_, and *N*-phenylmaleimide-mediated thermal closure. Notable, are the improved syntheses of H_2_@C_60_ (95 mol% H_2_) and ^4^He@C_60_ (50 mol% ^4^He), syntheses of HD@C_60_ (83 mol% HD) and ^3^He@C_60_ (52 mol% ^3^He) despite the commercial availability of HD and ^3^He at only low-pressure, and the first synthesis of Ne@C_60_ (63 mol% Ne—enriched to 100 mol% by recycling preparative HPLC)^73^.

#### Synthesis of closed fullerenes containing larger noble gas atoms or small molecules

The 16-membered cage-opening of **31** (or **40**) is too small to achieve entry of molecules larger than H_2_O, or of noble gas atoms larger than neon.

**Fig. 7:**
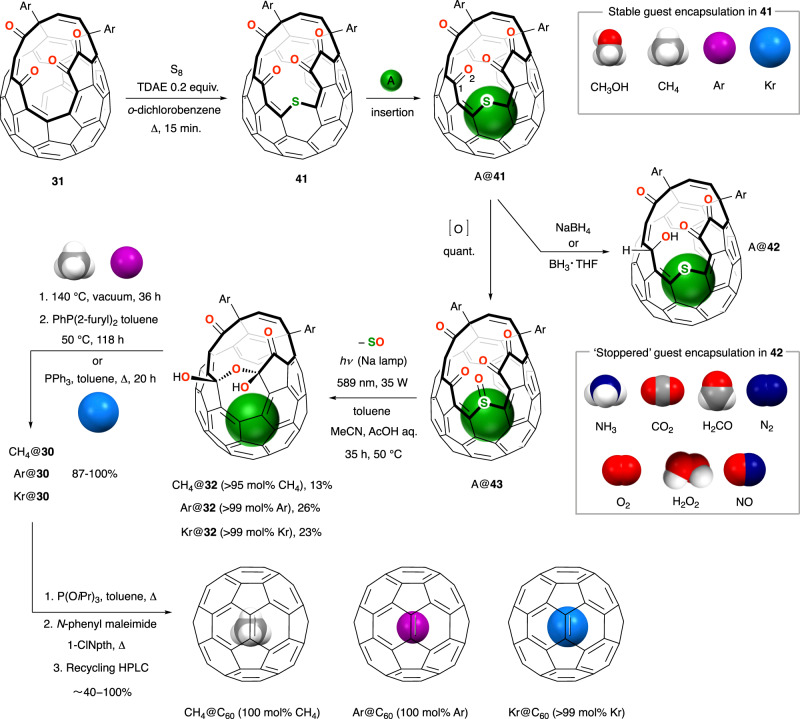
Synthesis of C_60_ endofullerenes containing a large endohedral species. **a** Open-cage fullerene **41** has a 17-membered orifice able to accommodate the entry of large guest species. Conditions for encapsulation of the guest species ‘A’ are described in the main text. Stable host-guest complexes CH_4_@**41**, Ar@**41** and Kr@**41** are intermediates in the synthesis of CH_4_@C_60_, Ar@C_60_ and Kr@C_60_; but more labile guests are characterised in the ‘stoppered’ open-fullerene A@**42**. Conditions for cage-closure of A@**42** have not yet been developed. 1-Chloronaphthalene (1-ClNpth), the ‘Ar’ 5-*tert*-butylpyridyl substituent structure is shown in Fig. 5a.

However, the encapsulation of ‘large’ molecules CO, NH_3_ and CH_4_ into Iwamatsu’s 17-membered cage-opened C_60_ derivative **15** (Fig. 2) encouraged Yasujiro Murata to apply the sulfur insertion method that he and Komatsu had earlier developed (for widening the orifice of **22** to **25**, Fig. 3) for expansion of the opening of **31**. Insertion of a sulfur atom into the rim of **31** was achieved using S_8_ in the presence TDAE to afford **41**, which has a 17-membered opening (Fig. 7)^84^. Rapid exchange of water in/out of **41** at room temperature in CDCl_3_ indicates the opening to be larger than that of **15**—thermal dissociation of H_2_O@**15** is relatively slow^51^ —and in order that encapsulation of large species ‘A’ by **41** is a viable route for the synthesis of closed endofullerenes A@C_60_, it is obviously necessary that sulfur extrusion to contract the cage-opening (returning to A@**31**) can be performed using conditions under which the endohedral species is not lost from A@**41**, or from an intermediate in the process of sulfur removal. Development of conditions for synthesis of A@C_60_ *via* routes that rely upon encapsulation into **41** has therefore required knowledge of the energetics of the encapsulation and loss, A + **41** ⇌ A@**41**.

So, Murata demonstrated pressure-dependent insertion of CH_3_OH into **41** in chlorobenzene solution at 150 °C, achieving up to 60 mol% encapsulation of CH_3_OH at 9000 atm, and also noting that contamination of the CH_3_OH@**41** product with N_2_@**41** indicates that partial solubilisation of gaseous species enables their insertion under pressure^85^. Accordingly, Whitby achieved 65 mol% encapsulation of CH_4_ into **41** in 1-chloronaphthalene solution at 200 °C, under 153 atm of methane^86^. Both CH_3_OH@**41** and CH_4_@**41** are stable at room temperature, showing no loss of the endohedral molecule over many months, and confirmed by the experimental kinetic parameters for thermal dissociation of CH_4_@**41**; *E*_a_ = 134.6 ± 5.0 kJ mol^−1^ and Δ*G*^‡^ = 151.5 ± 0.1 kJ mol^−1^ at 165 °C. In contrast, insertion of formaldehyde (from 1, 3, 5-trioxane in chlorobenzene solution under 8000 atm, at 150 °C) gave H_2_CO@**41** with 35 mol% H_2_CO, but more than half of the H_2_CO is lost from a solution of H_2_CO@**41** in CDCl_3_ after 30 h at room temperature. To prevent the escape of H_2_CO from the cage, selective reduction of one carbonyl group C(1)-O(2) from the *exo*-face acts to ‘stopper’ the opening (Fig. 7), and the alcohol product H_2_CO@**42** suffers no loss of formaldehyde after many months of storage at room temperature^85^. The calculated free energy for entry of ammonia into **41** (62.3 kJ mol^−1^) indicates facile entry under ambient conditions, such that solution-phase exposure of **41** to methanolic ammonia under dry conditions (to avoid encapsulation of water) results in rapid formation of NH_3_@**41**, but the complex is unstable to loss of NH_3_ similarly to the instability of H_2_CO@**41** to loss of formaldehyde, and cannot be isolated. Instead, in situ reduction of NH_3_@**41** using NaBH_4_ affords NH_3_@**42** with >90 mol% NH_3_ incorporation^86^. In recent years, several examples of this ‘stoppered’ open fullerene A@**42** have been obtained by encapsulation of guests with a low energy barrier to escape from **41**, followed by reduction with NaBH_4_ or BH_3_･THF; N_2_@**42** (43 mol% N_2_)^87^, CO_2_@**42** (76 mol% CO_2_)^87^, ^3^O_2_@**42** (81 mol% ^3^O_2_)^88^, NO@**42** (90 mol% NO)^89,90^ and H_2_O_2_@**42** (35 mol% H_2_O_2_)^91^. In each case, samples of pure A@**42** can be obtained by recycling preparative HPLC—with the exception of H_2_O_2_@**42** whose complete separation from contaminant H_2_O@**42** is laborious.

Earlier we described the first steps for repair of the cage-opening of Komatsu’s sulfide, **25**, via oxidation to the corresponding *exo*-sulfoxide **28**, then photochemical desulfinylation under visible light irradiation ((Fig. 4), and it is straightforward to envisage these key steps applied to contract the cage-opening of **41** (i.e., more importantly, A@**41**). Indeed, oxidation of **41** using dimethyl dioxirane (DMDO) or *m*CPBA cleanly furnishes *exo*-sulfoxide **43** without trace of the *endo*-sulfoxide or sulfone, but upon attempted photodesulfinylation of a mixed sample of N_2_@**43**, H_2_O@**43** and empty **43** using visible irradiation (Xe lamp, benzene, room temp., 21 h)— i.e., conditions comparable to those reported for elimination of SO from H_2_@**28** and ^4^He@**28**—the anticipated desulfinylation product(s) N_2_@/H_2_O@/empty **31** were not obtained^92^. Nonetheless, mass spectrometric analysis of **43** implied that SO extrusion is feasible; a peak corresponding to [M+H − SO]^+•^ appears in the atmospheric pressure chemical ionisation (APCI) mass spectrum^92^ and, significantly, the radical cation [M − SO]^+•^ is the dominant species in the atmospheric pressure photoionisation (APPI) mass spectrum^93^. Encouraged to pursue the possibility of photo-induced desulfinylation of **43**, and noting that the expected product of desulfinylation **31** is unstable under visible light irradiation, Bloodworth and Whitby found that the reaction was facilitated by trapping **31** as its more photo-stable bis(hemiketal) hydrate **32** in situ. CH_4_@**43** was prepared with >99.5 mol% CH_4_ content by heating powdered fullerene **41** at 190 °C under >1500 atm of methane, before oxidation with DMDO; then, under irradiation at 589 nm with a low-pressure sodium lamp for 35 h in a mixed solvent system of toluene, acetonitrile and acetic acid (10% *v*/*v* aq.), CH_4_@**43** successfully underwent loss of SO and hydration to give CH_4_@**32**—although in only 13% isolated yield. The <1% content of ‘empty’ **43** carried through this reaction encapsulates water under the aqueous conditions such that a trace of H_2_O@**32** contaminates the CH_4_@**32** product, so endohedral water is removed at 140 °C under a dynamic vacuum (conditions that also effect dehydration of CH_4_@**32** to CH_4_@**31**) without loss of methane, before completion of the final cage closure steps. The first of the two sequential reductive couplings (of A@**31**, then A@**30**, see Fig. 5b and the earlier discussion) was carried out using PhP(2-furyl)_2_ at 50 °C, i.e., under the mild conditions originally developed to attenuate loss of HF during reduction of HF@**31** (Fig. 6), but now because the temperature is too low for re-entry of water traces. The second reduction (of CH_4_@**30**) was safely achieved with P(O*i*Pr)_3_ in refluxing toluene as the opening of **30** is too small to accommodate water, and the final *N*-phenylmaleimide-mediated closure step gave pure CH_4_@C_60_ after removal of the traces (<1%) of empty C_60_ by recycling HPLC (Fig. 7)^93^.

The successful (if low-yielding) photo-desulfinylation of CH_4_@**43** has also enabled the method to be applied for preparation of the larger noble gas endofullerenes, Ar@C_60_^94^ and Kr@C_60_^95^ (Fig. 7). DFT calculations of the barrier to entry and binding enthalpies for encapsulation of the larger noble gas atoms argon and krypton by **41**, cf. methane, indicated that filling could be achieved under similar high-pressure conditions. Accordingly stable open endofullerenes Ar@**41** and Kr@**41** were obtained with near-quantitative incorporation of the noble gas atom under conditions similar to those that gave >95 mol% CH_4_ encapsulation: ca. 1400 atm of argon, or *ca*. 1500 atm of krypton, at 180 °C. Completion of the syntheses of Ar@C_60_ and Kr@C_60_ was carried out according to the methods described for CH_4_@C_60_, with improved isolated yields of 26% and 23% of Ar@**32** and Kr@**32**, respectively, from the key photo-desulfinylation step, suggesting that larger endohedral species’ inhibit the reaction. However, experiments with mixed CH_4_@**43**/H_2_O@**43** samples cannot distinguish between an inhibitory effect of methane and a promoting effect of water for example^93^, and the mechanism by which an endohedral species influences fullerene reactivity in the desulfinylation step remains to be fully understood. As methanol is a larger species than methane but has an electronic structure closer to water, it is of interest to note that of the group of stable complexes A@**41** given in Fig. 7, it is the earliest reported example CH_3_OH@**41** that remains an unused intermediate, i.e., the synthesis of CH_3_OH@C_60_ has not yet been pursued to our knowledge.

### Outlook

The synthesis of noble gas endofullerenes He@C_60/70_, Ne@C_60_, Ar@C_60_ and Kr@C_60_, molecular endofullerenes H_2_@C_60/70_, (H_2_)_2_@C_70_, HF@C_60_, H_2_O@C_60/70_, (H_2_O･HF)@C_70_ and CH_4_@C_60_, and isotopologues of several of these, are significant achievements from the research groups of Koichi Komatsu, Yasujiro Murata and Richard Whitby.

The dominant open-fullerenes now employed as key intermediates for guest encapsulation in molecular surgery are **31** (16-membered opening for entry of ‘small’ atoms and molecules), and **41** (17-membered opening for entry of larger species). The classification of endohedral guest species’ as ‘small’ or ‘large’ is not intended to imply that encapsulation depends solely upon their size, as the energies of activation for guest entry/exit and binding inside the cage depend on both steric and electronic interactions. Rather, this grouping reflects a calculated barrier to encapsulation into **31** that informs the authors’ own work.

Many studies of the properties of non-metal endofullerenes have been facilitated by the availability of the materials, although a review of these is sadly beyond our scope here. Similarly, theoretical study of endofullerenes is a very large field and our own motivation for endofullerenes synthesis is both the opportunity for their direct study and also the value of resulting data as a test of theoretical models. To satisfy these needs, many synthetic challenges remain to be addressed.

A low-yielding photochemical ring-contraction step is a constraint of the current method for synthesis of A@C_60_ via A@**41**, limiting the yield and material quantities which can be obtained when A is ‘large’ (CH_4_, Ar, or Kr to date). An understanding of the mechanism and approaches to optimisation of this limiting step, are of great importance—not only to overcome the low yield, but also to inform new routes for ring-closure. Two major targets, not yet achieved, are synthesis of NH_3_@C_60_ and O_2_@C_60_, which have exciting applications in nuclear hyperpolarisation. These species cannot be accommodated by the smaller cage-opening of **31** but escape rapidly from the larger opening of **41**.

A ‘stoppered’ open-fullerene **42** restricts the escape of NH_3_ and O_2_ (as well as N_2_, NO, CO_2_, H_2_CO and H_2_O_2_) but does not, in our hands, undergo contraction of the cage-opening under similar conditions to the photo-desulfinylation of A@**43**.

Noble gas endofullerenes are of enormous contemporary interest as the first series of compounds in which it is possible to study internuclear interactions between a noble gas atom and the cage, or in the noble gas dimer. Encapsulation of xenon by **41** is calculated to have Δ*H*^‡^_entry_ = 152 kJ mol^−1^ and Δ*H*^bind^ = −56 kJ mol^−1^, a significantly higher barrier to encapsulation than for krypton (Δ*H*^‡^_entry_ = 87 kJ mol^−1^ and Δ*H*^bind^ = −57 kJ mol^−1^)^95^, the largest noble gas encapsulated in an open-fullerene so far. In consequence attempted preparation of Xe@**41** under >1800 atm pressurisation of xenon gas, at 212 °C for 17 h, results in negligible (<1%) xenon incorporation^95^, restricting the range of noble gas endofullerenes available for study. Furthermore, the possibility of encapsulating still larger guests (including dimers) in the bigger cavity of C_70_ has not been realised, in part because a C_70_ derivative with the ‘large’ 17-membered opening corresponding to the structure of **41**, remains elusive^80,96^.

Solutions to these challenges, e.g., involving new ring-closure methods and alternative ‘large’ cage-opened derivatives of C_60/70_, informed by both experimental and computational studies, is where much effort is currently directed in the field.

## Supplementary information

Bloodworth_PR File
