# Supplementary material for: Synthesis of endohedral fullerenes by molecular surgery
Source: Commun Chem. 2022 Oct 8;5:121. doi: 10.1038/s42004-022-00738-9 (PMC9814919; doi:10.1038/s42004-022-00738-9)
Supplement: Supplementary file 1 — Bloodworth_PR File [file 42004_2022_738_MOESM1_ESM.pdf]

nature portfolio

Peer Review File

Synthesis of endohedral fullerenes by molecular surgery

Open Access This file is licensed under a Creative Commons Attribution 4.0 International License, which permits use, sharing, adaptation, distribution and reproduction in any medium or format, as long as you give appropriate credit to the original author(s) and the source, provide a link to the Creative Commons license, and indicate if changes were made. In the cases where the authors are anonymous, such as is the case for the reports of anonymous peer reviewers, author attribution should be to 'Anonymous Referee' followed by a clear attribution to the source work. The images or other third party material in this file are included in the article's Creative Commons license, unless indicated otherwise in a credit line to the material. If material is not included in the article's Creative Commons license and your intended use is not permitted by statutory regulation or exceeds the permitted use, you will need to obtain permission directly from the copyright holder. To view a copy of this license, visit http://creativecommons.org/licenses/by/4.0/.

REVIEWERS' COMMENTS:

Reviewer #1 (Remarks to the Author):

Entrapment and manipulation of single-molecules is an exciting and rapidly developing area of physical sciences. Endohedral fullerenes with a molecule (or atom) being incarcerated within the cavity of a carbon cage are at the forefront of this area. Molecular entrapment opens up numerous opportunities to study fundamental chemistry, structure and dynamics of isolated molecules, with many examples based on endohedral fullerenes. The key challenge here is the chemical process of opening the cage, insertion of guest-molecules followed by sealing the cage off. It requires high-level synthetic effort, including innovative methods and reagents of organic synthesis developed specifically for fabrication of endohedral fullerenes, termed collectively as the 'molecular surgery' approach. The authors of this article are internationally leading in the field, with many ground-breaking innovations accomplished at Southampton. The review article gives a historical perspective on molecular surgery with fullerenes as well as an excellent discussion of key experimental procedures, rich in detail and giving a sense of journey through 32 years of fullerene chemistry. I find this article quite inspiring, timely and useful.

The text is well-written and illustrated with high-quality ChemDraw diagrams allowing the reader to appreciate the challenges and opportunities of this area. All relevant references are given. The logical flow is coherent and writing style is clear and accessible to general readership. The paper can be published essentially as it is. I would invite the authors to consider the following points:
1) Could the meaning of 'small' and 'large' endohedral molecule be quantified (even approximately) from the viewpoint van der Waals diameter / critical dimension?
2) Diagram in Fig 1a (Rubin's two-step saturation method) could benefit from H-atoms being shown explicitly.
3) The authors are known for effective use of DFT calculations to guide the choice of reagents best sealing off the cage (phosphines), for example. I think that there is an opportunity to elaborate on the role of computation chemistry in the development of 'molecular surgery'.
4) If space permits, it would be really useful to include a paragraph on spectroscopy / microscopy of endohedral molecules. There are some excellent works (including the authors) that would be good to cite and discuss briefly to emphasise the importance of these materials.

Overall, this is an excellent and highly original contribution which should be published.

Reviewer #2 (Remarks to the Author):

In this review article Bloodworth and Whitby provide an overview on the synthesis of non-metal-encaged endohedral fullerenes via open-cage intermediates. Particularly, this review is focused on the synthesis of closed endofullerenes. The studies on such so-called molecular surgery of fullerenes have been conducted by a limited number of organic chemists, however, the unique structures and the properties are undoubtedly valuable to a broad readership of chemists. Thus, I would like to recommend its publication in Communications Chemistry after minor revisions suggested below.
1. For another example, the effect of a nitrogen atom encapsulation upon the chemical reactivity of the fullerene cage was also reported, see Chem. Commun. 2003, 2940-2941.
2. Isolation and characterization of N@C60 was reported in 2010, see: Chem. Comm. 2010, 46, 631-633.
3. It seems to be not easy for the readers to understand the orifice size of each open-cage intermediate. In this respect, the use of space-filling model to display the orifice size may be helpful.
4. In the schemes, extremely high pressure conditions have been applied to accomplish the encapsulations. At this point, what kind of a special apparatus was required to do that? Additional explanation of the special apparatus could improve the readability.

Reviewer #3 (Remarks to the Author):

Synthesizing closed cage fullerenes with entrapped atoms or molecules has recently been developed by using the method of molecular surgery, such as He@C60、H2O@C70. Molecular surgery is a very challenging task to synthesize endohedral fullerenes through methods of organic chemistry. This article reviews the current achievements of open-cage fullerenes, including cage-opening and closure methods, and the synthesis of non-metallic endohedral fullerenes by molecular surgery. The two main synthetic routes for encapsulation are ‘small’ guest species He, Ne, H2, HF or H2O and all larger noble gas atoms and small molecules. Synthesizing endohedral fullerenes by molecular surgery is of great significance for the spectroscopic study of the quantized energy level structure of the trapped species, the interactions between trapped species and carbon cages, and the effects of an encapsulated species upon the properties and reactivity of the cage. I recommend publication after minor revisions considering the following comments.
1. Whether can some straightforward examples be provided for the application of endohedral fullerenes created through molecular surgery? Are there some specific rules for its applications?
2. Two structures require modification. The two [5,6] bonds of the structure 4 in figure 1a should be solid lines, and the carbon atoms on two hexagons next to the opening hole designated as follows are wrongly bonded.

3. Figure 4b needs to add structure diagrams for H2@C70 and (H2)2@C70.
4. The full name should be used when the acronym THF appears for the first time in this article.
5. I suggest that the methods for cage opening can be tabulated by different precursors or synthesis methods.

Synthesis of endohedral fullerenes by molecular surgery

Response to Referees

Reviewer #1

1. Could the meaning of 'small' and 'large' endohedral molecule be quantified (even approximately) from the viewpoint van der Waals diameter / critical dimension?

The terms 'small' and 'large' which we use to indicate the two groups of guest atom/molecule that can be accommodated by open-fullerenes **31** and **41** respectively, cannot be quantified using a measure of the dimensions/size of the guest because the encapsulation of the guest is governed by not only steric, but also electronic interactions. We make this point with the following example in the main text:

*"The calculated activation energy for entry of H_2 into the cavity of **31** is ca. 12 kJ mol^{-1} higher than that for entry of H_2O[83], despite the smaller size of H_2 and presumably due to the attractive dipolar interactions of H_2O in the cage entrance."*

The only meaningful way to quantify the terminology 'small' and 'large' would therefore be to compare the energetics of guest encapsulation. Our own publications frequently cite the calculated energies of activation for guest entry and binding inside the cage, for specific comparative examples – however a tabulation of data for encapsulation of all the guest endohedral species' discussed in this review is not possible. The calculation methods differ between research groups, and the published literature is incomplete. We do have our own comprehensive calculations that inform our own research, but this is an unpublished dataset that is not suitable to cite, as the methods have not yet been peer-reviewed.

The existing text delineates that 'small' and 'large' are comparative terms based on experimental data, so we believe the distinction is clear from attentive reading of the text.

However, we have provided clarification to our concluding remarks as follows:

Original: *"The dominant open-fullerenes now employed as key intermediates for guest encapsulation in molecular surgery are **31** (16-membered opening for entry of 'small' atoms and molecules), and **41** (17-membered opening for entry of larger species)."*

New: *"The dominant open-fullerenes now employed as key intermediates for guest encapsulation in molecular surgery are **31** (16-membered opening for entry of 'small' atoms and molecules), and **41** (17-membered opening for entry of larger species). The classification of endohedral guest species' as 'small' or 'large' is not intended to imply that encapsulation depends solely upon their size, as the energies of activation for guest entry/exit and binding inside the cage depend on both steric and electronic interactions. Rather, this grouping reflects a calculated barrier to encapsulation into **31** that informs the authors' own work."*

2. Diagram in Fig 1a (Rubin's two-step saturation method) could benefit from H-atoms being shown explicitly.

We carefully considered this point when preparing Fig. 1, and the referee is correct to suggest that showing the position of the saturating substituents on **2** could assist the reader in grasping the chemical concept of Rubin's route more quickly (not necessarily hydrogen atoms though, other substituents would saturate the ring shown in bold for Rubin's purpose of achieving [2+2+2] opening). However, to show saturating substituents (*e.g.* hydrogen) requires the image to become very cluttered and, more seriously, the groups cannot be positioned on structure **3** without highly distorted bond angles. The image below shows how the proposed change would look:

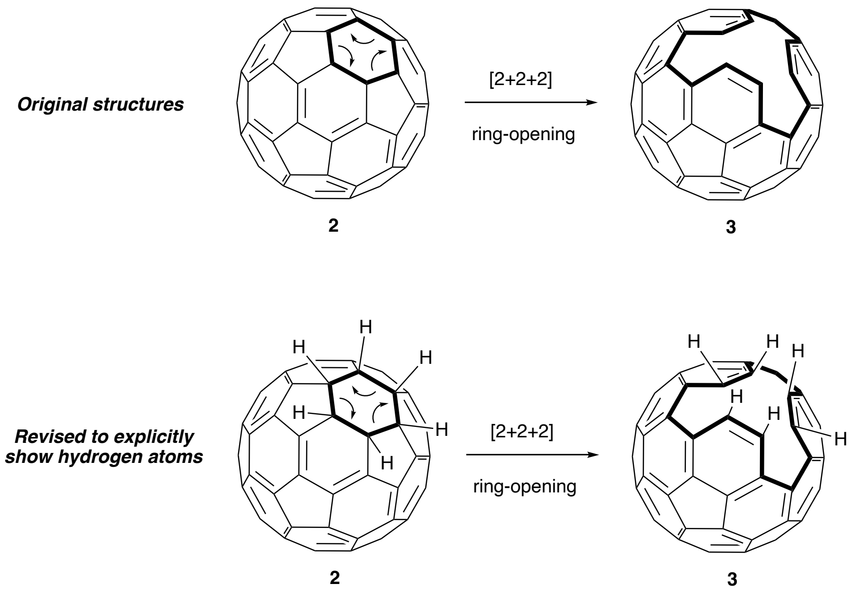

So, we prefer to retain the original image; to avoid cluttering and the poor-quality of revised structure **3**, and to avoid the suggestion that only hydrogen substituents are implied.

Instead, we have added the label '(saturating substituents not shown)' explicitly within the scheme. The absence of saturating substituents is already defined in the figure legend.

3. The authors are known for effective use of DFT calculations to guide the choice of reagents best sealing off the cage (phosphines), for example. I think that there is an opportunity to elaborate on the role of computation chemistry in the development of 'molecular surgery'.

Similarly to comment 4., below (requesting additional coverage of the spectroscopy of endofullerenes) the referee makes a good point, and if space had allowed we would have liked to provide more detail of how we use calculations to design synthesis of endofullerenes in our own group.

Instead, we have integrated examples throughout the text. Space constraints do not allow us to elaborate, and we believe to do so in the absence of some coverage of the supporting literature from other groups (well over 200 papers) would be a mistake and is clearly impracticable. As indicated below, we are planning a separate review which will include coverage of our theoretical work.

4. If space permits, it would be really useful to include a paragraph on spectroscopy / microscopy of endohedral molecules. There are some excellent works (including the authors) that would be good to cite and discuss briefly to emphasise the importance of these materials.

Indeed, we originally hoped to include some coverage of the spectroscopy of endohedral fullerenes. However, as the article developed it became clear that the available space only allowed for a concise coverage of synthesis, and our final preference was to exclude spectroscopy altogether: (i) in order not to compromise our coverage of the synthesis which fits the available word/space limits nicely and (ii) since a very limited coverage of spectroscopy would be so weak as to be almost meaningless. Even our own work in this area (commented on by the referee) runs to >20 papers, and there are dozens more by other authors. Our revised decision to exclude a specific section dedicated to spectroscopy in this review was made with agreement from the Editor before submission (since the article was invited) and we are planning a separate review of the spectroscopy and theoretical studies of endofullerenes for the near future instead.

Reviewer #2

1. For another example, the effect of a nitrogen atom encapsulation upon the chemical reactivity of the fullerene cage was also reported, see Chem. Commun. 2003, 2940-2941.

The reference suggested here by reviewer #2 details the influence of an endohedral nitrogen atom upon the photochemical reactivity of $N@C_{60}$ towards disilirane.

The influence of endohedral species upon the reactivity of C_{60} is important among the many applications we cite for these materials in our introduction text:

"... for study of the effect of an encapsulated species upon the properties and reactivity of the cage[10-13],"

So, we have added the suggested reference to the three citations previously given (new Ref. 10).

2. Isolation and characterization of N@C60 was reported in 2010, see: Chem. Comm. 2010, 46, 631-633.

We thank the referee for this important comment, as the suggested reference had escaped our notice. This paper describes isolation of pure $N@C_{60}$ ("several tens of μg") from a mixed sample of $N@C_{60}$ + C_{60} + $C_{60}O$ by recycling HPLC. We have added the reference (new Ref. 30) and modified our introduction text as follows:

Original: *"Again, the encapsulation level after exhaustive HPLC enrichment (<1%) and material yield (microgram scale) from these direct insertion methods is too low for many spectroscopic applications[29,30]."*

New: *"Pure $N@C_{60}$ has been isolated by exhaustive HPLC enrichment,[30] although the material yield (microgram scale) from these direct insertion methods is too low for many spectroscopic applications[30-32]."*

3. It seems to be not easy for the readers to understand the orifice size of each open-cage intermediate. In this respect, the use of space-filling model to display the orifice size may be helpful.

In response to reviewer #1 (point 1.) above, we have clarified that encapsulation of the guest is governed by steric *and* electronic interactions during entry, as well as the binding energy inside the cage.

The use of space-filling models to display the orifice could suggest that the *size* of the opening is the only relevant factor. Although size is a dominant consideration, we believe that the detailed explanation of the encapsulation/escape equilibrium given in the text for individual open-fullerenes is appropriate. In order to determine if space-filling models would be a helpful visual aid we have prepared the image below of the two key open-fullerenes **31** (narrow opening, shown left) and **41** (wider opening, shown right) viewed from above the orifice.

However, we conclude that very little information is conveyed (and at the expense of a more nuanced understanding). We thank the referee for this suggestion but, after consideration, we choose not to include the image.

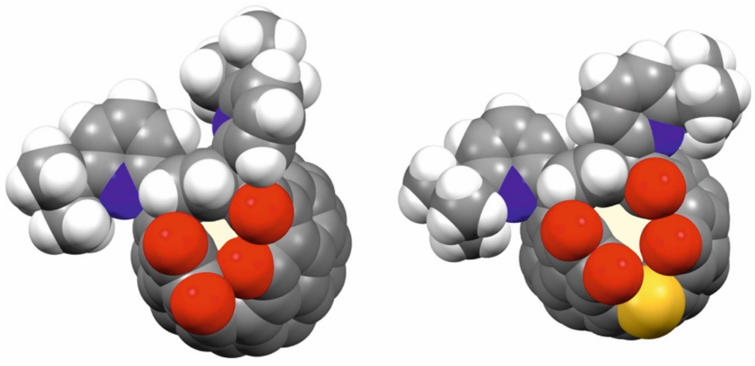

4. In the schemes, extremely high-pressure conditions have been applied to accomplish the encapsulations. At this point, what kind of a special apparatus was required to do that? Additional explanation of the special apparatus could improve the readability.

Examples of high-pressure reactions cited in the schemes (and text) of <500 atm or *ca.* <20 MPa (*i.e.,* all those in Figs. 1 and 2) are conditions that are accessible using ordinary Parr® high-pressure vessels, widely used in many organic chemistry laboratories. These conditions do not warrant special description.

The referee is correct to highlight that the extremely high-pressure conditions of >1500 atm. used in our own work, and cited in Fig.6, require bespoke apparatus. This apparatus is described in detail in the supplementary material of Ref. 73, so the following clarification has been added to the Fig. 6 legend:

"High-pressure solid-state filling was carried out in a 100 × 5.2 mm 316L stainless steel reactor as part of a bespoke apparatus for gas compression using a manual pump.[73] *"*

The apparatus used by other researchers to achieve similar high-pressure in the encapsulation procedures that we cite in the review also relies on gas compression, so at the first point where very high-pressure conditions are cited in the main text (helium encapsulation under 1230 atm) we have added the following statement:

"Encapsulation of endohedral species (e.g., ^{4}He in this example) under high-pressure conditions well in excess of 1200 atm is cited in many further examples, vide infra, and although we refer the reader to the original literature for details of the bespoke apparatus used therein, typically relies upon hydraulic or manual gas compression following initial pressurisation."

This explanation serves to improve the reader's understanding of the procedures involved without introducing an inappropriate level of experimental detail.

Reviewer #3

1. Whether can some straightforward examples be provided for the application of endohedral fullerenes created through molecular surgery? Are there some specific rules for its applications?

The referee here makes a similar comment to reviewer#1 (point 4) in noting that we have not given detailed coverage of the applications of endohedral fullerenes. There are not specific rules for the applications of endohedral fullerenes which can be stated concisely, as this is a very large field including study of the quantised behaviour of trapped species using NMR, IR and THz spectroscopy or inelastic neutron scattering, nuclear spin isomerism and its macroscopic effects, endohedral species as NMR probes, the effect of endohedral species upon photochemistry, upon mechanical properties and upon chemical reactivity of fullerenes, and endofullerenes as tools for validation of theoretical models. As indicated in our response to reviewer#1 (above) it is not feasible to cover this material concisely within this review.
Our introduction text includes the following paragraph:

"They are compounds of enormous interest in several areas; for spectroscopic study of the quantised energy level structure of the trapped species[4-7], for study of the internuclear (host-guest) interactions resulting from encapsulation and validation of predictive models of these interactions[8,9], for study of the effect of an encapsulated species upon the properties and reactivity of the cage[10-13], and for the materials applications that arise in each of these areas."

such that an interested reader is directed to the previous reviews in this area (clustered in the *Philos. Trans. R. Soc., Ser. A* **371** special issue of 2013) and some key references that highlight the most significant recent developments in the listed fields of study.
A separate, new review of the spectroscopy and theoretical studies of endofullerenes is planned by our own group.

2. Two structures require modification. The two [5,6] bonds of the structure 4 in figure 1a should be solid lines, and the carbon atoms on two hexagons next to the opening hole designated as follows are wrongly bonded.

Corrected.
In our images we sometimes use 'grey' bonds to convey transparency to parts of the complex structures, as this usually gives more clarity. However, the referee is correct to notice that some grey bonds in structure **4** are integral to the chemistry taking place in **4**→**5** (Fig. 1b, not Fig. 1a) so we have made these bonds 'solid'.
An apparent mistake in bonding arose because the role of $^1O_2$ above the arrow in **4**→**5** includes an oxidation step that was not explicitly stated in the original figure. We have corrected this by clearly specifying the three steps on the arrow (1. Cycloaddition, 2. [2+2+2] ring-opening, 3. Oxidation).

3. Figure 4b needs to add structure diagrams for H2@C70 and (H2)2@C70.

In Fig. 4a, $^4He@C_{60}$ and $H_2@C_{60}$ are shown with full structural images as they are the first complete syntheses of closed-cage endofullerenes to appear in the article, which we suggest warrants the important visual impact of showing them.

The three additional product endofullerenes $He@C_{70}$, $H_2@C_{70}$ and $(H_2)_2@C_{70}$ shown in Fig. 4b are given without drawn structures, since the ‘$A@C_{60/70}$’ formula convention conveys all of the structural information.
The referee requests structural diagrams only for $H_2@C_{70}$ and $(H_2)_2@C_{70}$ (but not $He@C_{70}$) in Fig. 4b. Yet we have also repeatedly used only the formula to depict many products ‘$A@C_{60}$’ in Figs. 5 and 6., doing so in order to save space in the figures when structural diagrams would convey no additional useful information or clarity.
Furthermore, as $H_2@C_{70}$ and $(H_2)_2@C_{70}$ are distinct products in Fig. 4b and both would need to be shown to meet the referee’s request, there is insufficient space available.
We thank the referee for this suggestion, but we believe changes to Fig 4b. are unnecessary.

4. The full name should be used when the acronym THF appears for the first time in this article.

We have added ‘tetrahydrofuran (THF)’ to the abbreviations defined in the legend of Fig. 4.

5. I suggest that the methods for cage opening can be tabulated by different precursors or synthesis methods.

The referee has here highlighted one of the key challenges in writing this review – the ordering, sub-division and summary of the material.
We very carefully considered these points in arriving at the current ordering of the manuscript. To further tabulate the material under alternative criteria would require a lot of additional space, without justification for the re-ordering/repetition. In fact, the current logical progression of the content would be severely disrupted, and we note that the existing structure is particularly well-received by reviewer #1.
In this matter of individual preference, we do not believe there is an advantage to re-tabulating material that currently straddles different sub-sections as this would impose significant changes to the layout. We thank reviewer #3 for their suggestion, but no changes have been made.
